# Gas subcision with PDLLA (Juvelook): Evaluating a hybrid mechanical–biologic approach for atrophic acne scars

**DOI:** 10.1016/j.jpra.2026.05.001

**Published:** 2026-05-30

**Authors:** Kyu-Ho Yi, Isabella Rosellini, Suyeon Lee, Han Earl Lee

**Affiliations:** a You and I Clinic, Seoul, South Korea; b Avery Beauty Clinic And Avena Aesthetics Indonesia; c Medical Research Inc., Seoul, South Korea; d Opening Plastic Surgery Clinic, South Korea

**Keywords:** Carbon dioxide subcision, Atrophic acne scars, Dermal remodeling, Fibroblast activation, Collagen regeneration, Carboxytherapy, Minimally invasive scar treatment, Juvelook

## Abstract

**Background:**

Atrophic acne scars result from impaired dermal repair following inflammation of the pilosebaceous unit, leading to collagen degradation, dermal atrophy, and tethering by fibrotic strands. Conventional subcision mechanically releases these adhesions but provides limited biologic stimulation for dermal remodeling. Carbon dioxide (CO₂) gas subcision has emerged as a hybrid mechanical–biologic modality that integrates fibrotic release with CO₂ -induced vasodilation, microcirculatory enhancement, and fibroblast activation.

**Objective:**

To describe the clinical use and short-term outcomes of CO₂ gas subcision for atrophic acne scars and explore its potential as a minimally invasive hybrid treatment.

**Methods:**

CO₂ device (Trifill Pro, South Korea) and PDLLA (Juvelook, VAIM Inc., South Korea) was used in the study. A temperature-regulated medical-grade CO₂ system delivered controlled intradermal gas through a 30-gauge needle. Short CO₂ bursts were administered into the deep dermal or upper subcutaneous plane to detach fibrotic bands while inducing hypercapnia-associated biologic responses. Five patients with atrophic acne scars underwent a single session. Follow-up at weeks 2 and 4 included standardized photography, clinical assessment of scar depth and contour, and patient-reported satisfaction using a 0–10 Visual Analogue Scale (VAS).

**Results:**

Five patients completed follow-up at 4 weeks. All patients demonstrated qualitative improvement in scar depth and contour on clinical assessment. Mean patient satisfaction score (VAS) was high (range 7–9). No serious adverse events were observed. Mild transient erythema and edema resolved spontaneously within hours. Due to the small sample size and short follow-up, results are descriptive rather than statistically powered.

**Conclusions:**

CO₂ gas subcision appears to be a feasible and well-tolerated minimally invasive technique combining mechanical fibrotic release with biologic stimulation. This pilot case series suggests short-term improvement in atrophic acne scars; however, larger controlled studies with longer follow-up are required to confirm efficacy and durability.

**Evidence level:**

V

Blinded manuscript without author contact information.

## Introduction

Atrophic acne scars are a common sequela of acne vulgaris with significant aesthetic and psychological impact. Subcision is a well-established treatment for rolling scars, but outcomes may be limited by incomplete release and variable collagen remodeling. Carbon dioxide (CO₂) gas subcision represents a modified approach combining mechanical tissue separation with potential biologic stimulation through improved microcirculation and fibroblast activation.^1^, ^2^, ^3^, ^4^, ^5^

This pilot case series aimed to evaluate the short-term clinical outcomes and safety of CO₂ gas subcision in patients with atrophic acne scars. Given the increasing interest in minimally invasive and combination-based approaches for acne scar management, evaluating simplified techniques with dual mechanical and biologic effects is clinically relevant.

## Methods

### Study design and patient selection

This study was conducted as a prospective observational case series evaluating the effects of carbon dioxide (CO₂) gas subcision on atrophic acne scars. Five patients were included consecutively at a single clinical center. All treatments were performed in a single session.

### Inclusion criteria

Inclusion criteria were adults aged ≥18 years with clinically diagnosed atrophic acne scars (ice pick, boxcar, or rolling), stable acne, and Fitzpatrick skin types I–V. Exclusion criteria included active acne or infection in the treatment area, history of keloid or hypertrophic scarring, recent systemic retinoid use, coagulopathy, pregnancy, or any condition that could impair wound healing or increase procedural risk.

The study was conducted in accordance with ethical standards for human research. All participants provided written informed consent for the procedure and clinical photography. Due to the minimal-risk observational design and absence of identifiable data, the study qualified for institutional review board exemption according to local regulations

### Procedure

A medical-grade CO₂ device (Trifill Pro, Korea) delivered gas via a 30-gauge needle with digitally controlled flow with PDLLA (poly-D,L-lactic acid). PDLLA formulation: Juvelook (VAIM Inc., South Korea), 50 mg reconstituted in 10 mL normal saline. After skin preparation and topical anesthesia, the needle was inserted into the deep dermal or subcutaneous plane, and CO₂ was injected at multiple points (1–1.5 cm apart) to achieve tissue elevation and separation and Juvelook was injected along the CO2 emission.

### Post-procedure care

Post-procedure care included cold compress and avoidance of heat, massage, and sun exposure for 48 h.

### Outcome assessment

Patients were evaluated at 2 and 4 weeks using standardized photographs, clinical assessment of scar improvement, patient satisfaction (VAS 0–10), and adverse event monitoring.

## Results

Five patients (24–38 years; 3 females, 2 males; Fitzpatrick III–V) completed the study, all presenting with rolling and boxcar scars. At 4 weeks, all patients showed visible improvement in scar depth and contour. Patient satisfaction scores ranged from 7 to 9.

**Fig. 1:**
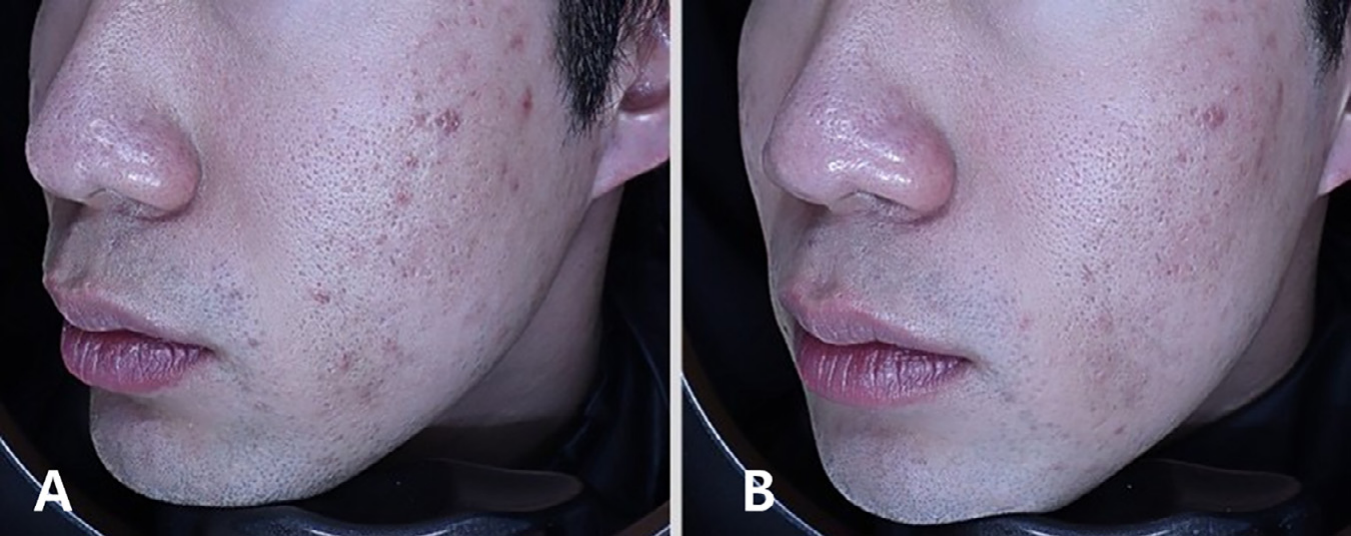
Patient with rolling atrophic acne scars on the cheek treated with CO₂ subcision. Clinical improvement in scar depth and contour is observed at 24 weeks post-treatment.

No serious adverse events occurred. Mild erythema and edema resolved spontaneously within hours. (Fig. 1)

## Discussion

This pilot case series suggests that CO₂ gas subcision may provide short-term improvement in atrophic acne scars with a favorable safety profile. The observed clinical improvements and high patient satisfaction are consistent with previous reports supporting gas-based subcision as a minimally invasive approach.^6^, ^7^, ^8^, ^9^, ^10^

The mechanism of action may involve both mechanical and physiologic effects. In addition to tissue separation, CO₂ injection may enhance local microcirculation and oxygen delivery, thereby promoting fibroblast activity and collagen remodeling as suggested in prior studies.^3^, ^4^, ^5^

These findings support the concept of gas-assisted subcision as a hybrid approach that may complement conventional techniques. Compared with traditional subcision, the addition of CO₂ may provide a more uniform tissue expansion and potentially reduce the need for more invasive adjunctive procedures.

From a clinical perspective, the minimally invasive nature of CO₂ gas subcision may offer advantages in terms of reduced downtime and patient tolerability compared with more aggressive resurfacing techniques. This may be particularly relevant for patients seeking gradual improvement with lower risk of post-inflammatory hyperpigmentation, especially in darker skin types.

In addition, the use of gas as a medium for subcision may allow more uniform tissue expansion compared with traditional needle-based techniques alone. This could potentially enhance the release of fibrotic bands in a controlled manner while minimizing tissue trauma, although this hypothesis requires further validation.

PDLLA (poly-D,L-lactic acid), as used in Juvelook-type formulations, supports dermal remodeling in atrophic acne scars by acting as a biodegradable collagen biostimulator rather than a simple volumizing filler. In acne scarring, dermal collagen is irregularly produced and degraded during wound healing, leading to depressed scars such as rolling, boxcar, and ice-pick scars. When PDLLA particles are delivered into the scarred dermis, they gradually stimulate fibroblast activity, extracellular matrix repair, and neocollagenesis; published PDLLA literature reports that collagen synthesis may begin around 6–8 weeks after injection, with type I collagen production continuing for 9–12 months. Histologic studies of injectable PDLLA have shown increased collagen and elastic fibers in the dermis, supporting its role in improving dermal thickness, elasticity, scar depression, and overall skin texture.^11^, ^12^, ^13^, ^14^, ^15^

Further studies with larger cohorts, objective outcome measures, and longer follow-up are required to confirm the efficacy and durability of this technique. These preliminary findings highlight the potential role of CO₂ gas subcision as an accessible adjunct or alternative within multimodal acne scar treatment strategies.

## Conclusion

CO₂ gas subcision is a minimally invasive technique for atrophic acne scars that may offer short-term clinical improvement with minimal adverse effects. These preliminary findings require confirmation in larger controlled studies with standardized assessment methods.

## Lay summary

In this small pilot series of 5 patients, CO₂ gas subcision showed short-term improvement in atrophic acne scars with only transient redness and swelling. The technique uses controlled CO₂ delivery through a fine needle to release scar bands and may potentially support collagen remodeling. These findings are preliminary and hypothesis-generating, and larger studies are needed.

## Informed consent

Informed consent was acquired.

## Funding

None.

## Ethical approval

The study was conducted in accordance with the Declaration of Helsinki.

## Author contributions

All authors have reviewed and approved the article for submission. Conceptualization, Kyu-Ho Yi, Isabella Rosellini, Suyeon Lee, Han Earl Lee

Writing—Original Draft Preparation, Kyu-Ho Yi, Isabella Rosellini.

Writing—Review & Editing, Kyu-Ho Yi, Suyeon Lee, Han Earl Lee.

Visualization, Kyu-Ho Yi, Isabella Rosellini, Suyeon Lee.

Supervision: Kyu-Ho Yi.

## Declaration of competing interest

None declared.

## Appendix Supplementary materials

Image, application 1
