# Supplementary material for: Gas subcision with PDLLA (Juvelook): Evaluating a hybrid mechanical–biologic approach for atrophic acne scars
Source: JPRAS Open. 2026 May 30;51:129–33. doi: 10.1016/j.jpra.2026.05.001 (PMC13321042; doi:10.1016/j.jpra.2026.05.001)
Supplement: Supplementary file 1 [file mmc1.docx]

**Supplementary Figures**


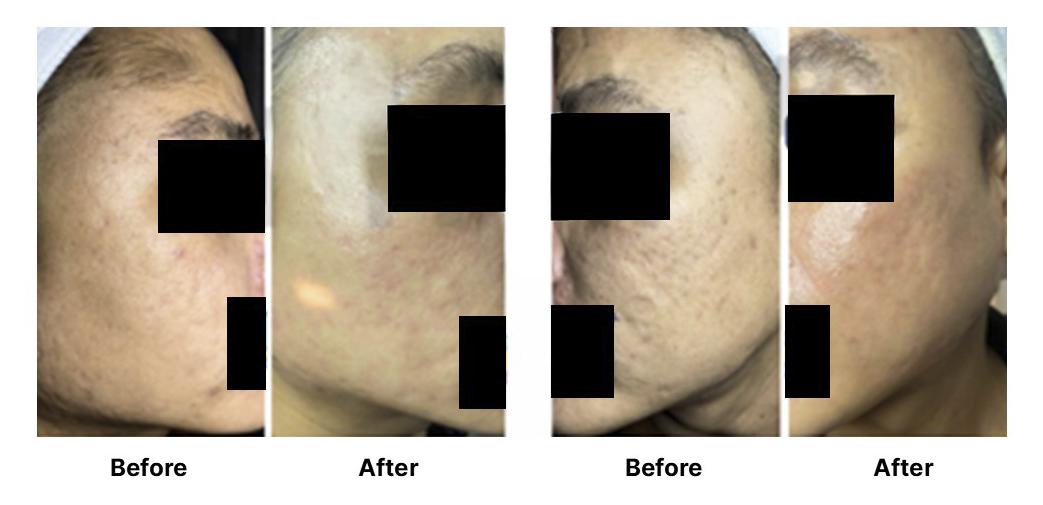


**Figure 2.** Patient with mixed rolling and boxcar scars on the lateral cheek. Visible reduction in scar depression is noted at 4-week follow-up.


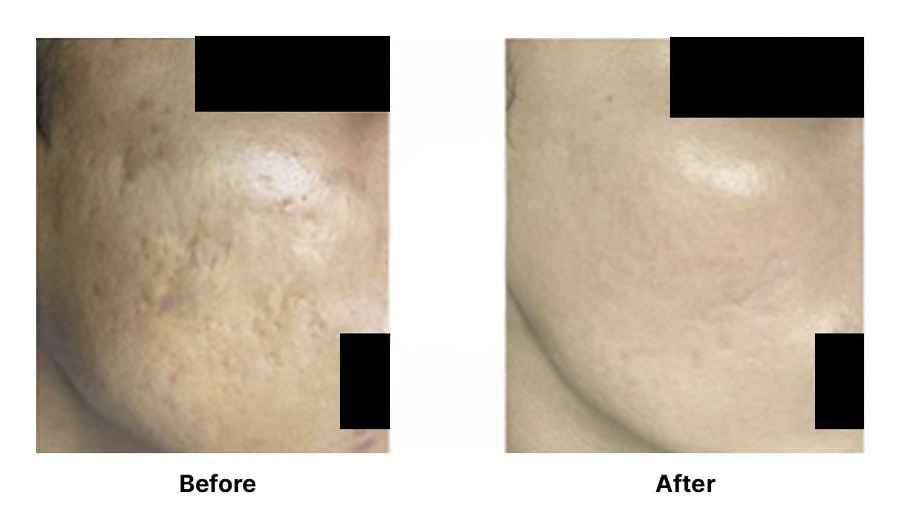


**Figure 3.** Patient presenting with boxcar scars on the bilateral cheeks. Post-treatment images at 4 weeks demonstrate qualitative improvement in skin texture.


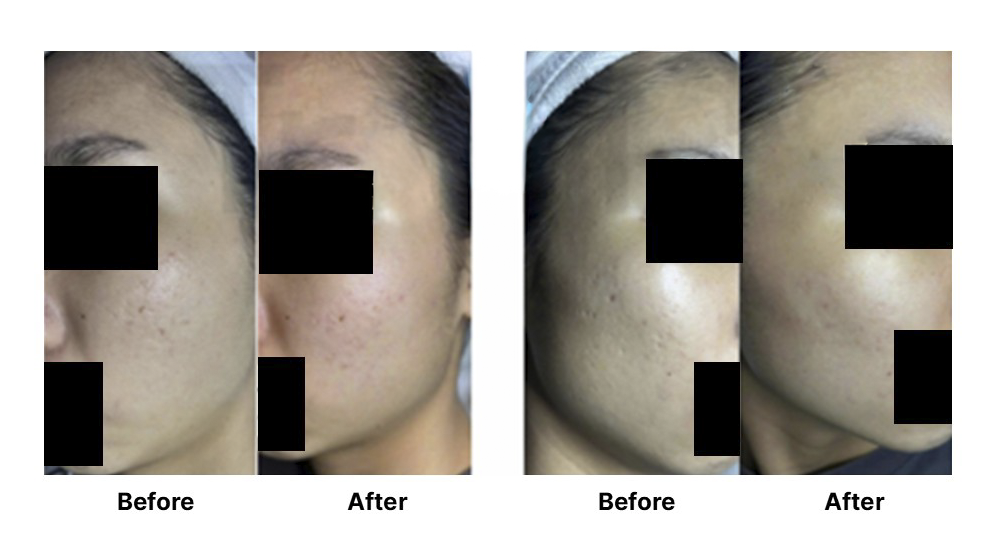


**Figure 4.** Patient with predominantly rolling scars in the malar region. Improvement in skin smoothness and contour is observed after one session at 4 weeks.


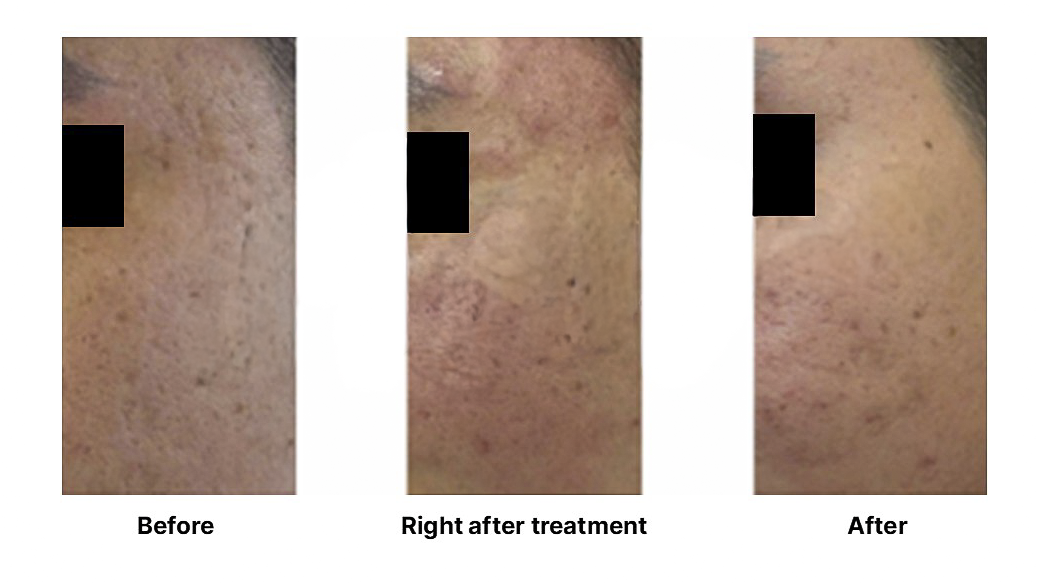


**Figure 5.** Immediate post-treatment appearance showing transient erythema following CO₂ subcision, with full recovery and improvement in scar appearance at 4 weeks.

**Supplementary Table 1. Individual patient characteristics and outcomes**

Note: The manuscript reports five patients aged 24–38 years, 3 females and 2 males, Fitzpatrick skin types III–V, rolling/boxcar atrophic acne scars, single-session CO₂ gas subcision, 2- and 4-week follow-up, VAS satisfaction range 7–9, and no serious adverse events. Patient-level fields should be verified against the original clinical record before production submission.

| **Patient** | **Age (years)** | **Sex** | **Fitzpatrick skin type** | **Predominant scar type** | **Treatment session** | **Follow-up completed** | **Clinical outcome at 4 weeks** | **VAS satisfaction (0–10)** | **Adverse events** |
| --- | --- | --- | --- | --- | --- | --- | --- | --- | --- |
| Patient 1 | To verify | To verify | III–V; specify | Rolling/boxcar; specify | Single CO₂ gas subcision session | 2 and 4 weeks | Visible qualitative improvement in scar depth/contour; specify | 7–9; specify | Mild transient erythema/edema if present; no serious adverse events |
| Patient 2 | To verify | To verify | III–V; specify | Rolling/boxcar; specify | Single CO₂ gas subcision session | 2 and 4 weeks | Visible qualitative improvement in scar depth/contour; specify | 7–9; specify | Mild transient erythema/edema if present; no serious adverse events |
| Patient 3 | To verify | To verify | III–V; specify | Rolling/boxcar; specify | Single CO₂ gas subcision session | 2 and 4 weeks | Visible qualitative improvement in scar depth/contour; specify | 7–9; specify | Mild transient erythema/edema if present; no serious adverse events |
| Patient 4 | To verify | To verify | III–V; specify | Rolling/boxcar; specify | Single CO₂ gas subcision session | 2 and 4 weeks | Visible qualitative improvement in scar depth/contour; specify | 7–9; specify | Mild transient erythema/edema if present; no serious adverse events |
| Patient 5 | To verify | To verify | III–V; specify | Rolling/boxcar; specify | Single CO₂ gas subcision session | 2 and 4 weeks | Visible qualitative improvement in scar depth/contour; specify | 7–9; specify | Mild transient erythema/edema if present; no serious adverse events |

Abbreviations: CO₂, carbon dioxide; VAS, Visual Analogue Scale.
